# Tackling Inequalities in Access to Medicines for People Experiencing Homelessness: A Meta‐Ethnography and Qualitative Systematic Review

**DOI:** 10.1111/hex.70076

**Published:** 2024-10-25

**Authors:** Tasnim Begum, Kylie Murrell, Anna Robinson‐Barella

**Affiliations:** ^1^ School of Pharmacy Newcastle University Newcastle upon Tyne UK; ^2^ Population Health Sciences Institute Newcastle University Newcastle upon Tyne UK

**Keywords:** access to medicines, homeless population, homelessness, inequality, medicines inequality, meta‐ethnography

## Abstract

**Introduction:**

Despite increased awareness of the detrimental relationship between homelessness and health, people experiencing homelessness remain an underserved population in health and social care research. Due to barriers affecting the accessibility of medicine and healthcare services, as well as reported competing priorities such as food and shelter, evidence has demonstrated that people experiencing homelessness are less likely to undergo routine examinations, receive diagnoses and adhere to prescribed medical treatments. To enhance service design and access for those experiencing homelessness, it is critical to better recognise, understand and address the barriers these individuals face. This meta‐ethnography aims to identify barriers, enablers and interventions to begin addressing this inequality gap.

**Methods:**

A systematic literature search was undertaken in October and re‐ran in December 2023 across four databases: MEDLINE, Embase, CINAHL and Scopus. Qualitative studies were included if they addressed barriers, enablers and interventions aimed at tackling medicines and health service inequalities among populations experiencing homelessness. Study quality was assessed using the Joanna Briggs Institute critical appraisal checklist. Data were synthesised using a meta‐ethnographic approach, as outlined by Noblit and Hare. The review was registered on PROSPERO (CRD42024511502) and performed according to PRISMA guidelines.

**Results:**

This meta‐ethnographic systematic review synthesised data from eight studies across multiple countries. Three overarching third‐order constructs (termed ‘themes’) were developed through reciprocal translation and centred around: recognising and acknowledging the discrimination, stigma and barriers experienced when using current services; exploring safe and practical use of medicines and the promotion of general health education and appreciating strategies to tackle inequalities, namely community outreach programmes designed for homeless communities.

**Conclusion:**

This work highlighted the barriers, enablers and interventions that sought to address the inequalities affecting people experiencing homelessness in accessing medication and healthcare services. Future research should utilise lived‐experience narratives and co‐design to further explore ways to tackle wider healthcare accessibility inequalities for this minoritised population.

**Trial Registration:**

Not applicable, as this is a systematic review.

**Patient or Public Contribution:**

Public contributors (minority research champions and one public health research champion, H.K.G. and T.G.) informed and shaped this project during study design and conceptualisation. They helped to ensure that the study was conducted, and the findings were reported with sensitivity.

## 1 Introduction

Over 100 million people experience homelessness worldwide each year, with consistent rises in the number of those who are either homeless or at risk of becoming so [1]. Homelessness has a significant influence on health in a number of ways, including close proximity to infectious diseases, inadequate nutrition, exposure to harsh conditions, reduced safety, poor hygiene and a general lack of rest and recuperation [2, 3, 4]. Due to barriers affecting their ability to access healthcare services and reported competing priorities such as food and shelter, evidence has demonstrated that people experiencing homelessness are less likely to undergo routine examinations, receive diagnoses and adhere to appropriate medical treatments [2, 5, 6]. Furthermore, studies have reported higher rates of multimorbidity, mental health disorders, substance abuse and social isolation among this vulnerable minority group [3, 7, 8, 9].

It has become evident that people experiencing homelessness are less likely to use mainstream primary care, given that the annual cost of unscheduled care is eight times higher than that of the housed population [10]. Specifically in the United Kingdom, people experiencing homelessness were reported to be four times more likely to visit acute hospitals for emergency care than the general population, but 40 times less likely to be registered with a general practitioner [11]. Recent research has demonstrated global inequalities in health outcomes experienced by those affected by homelessness [4, 12], attributing this to (i) barriers affecting their access to healthcare services and (ii) the absence of tailored health services to best meet their needs [13, 14, 15].

International government policies have sought to lower homelessness, enhance health outcomes and begin tackling the complexity of multimorbidity and inequality among this group. However, little research has been conducted on how best to put these initiatives into practice [16, 17]. Current research revealed the glaring disparity in health and quality of life between the homeless population and the housed population [14, 18]. The homeless population continues to be underserved despite the growing awareness of the detrimental link between homelessness and poorer health and medication outcomes [19]. To begin taking steps to close this inequality gap, and enhance healthcare services for those experiencing homelessness, it is critical to better understand, recognise and address the barriers these individuals face.

This meta‐ethnographic review aimed to synthesise existing qualitative research to facilitate a deeper understanding of the barriers, enablers and interventions seeking to address inequality among the homeless population when accessing medication and health services.

## 2 Methods

### 2.1 Protocol Registration

This meta‐ethnography and systematic review is registered with PROSPERO (registration number CRD42024511502) and has been conducted in accordance with the Preferred Reporting Items for Systematic Reviews and Meta‐Analyses (PRISMA) guidelines (Supporting Information S1: Appendix A) [20].

### 2.2 Eligibility Criteria

This meta‐ethnographic systematic review focused on addressing the barriers that people experiencing homelessness encounter when accessing medication and health services, as well as identifying enablers and interventions aimed to lessen the inequality gap. Therefore, to be included in this work, papers were required to meet the following eligibility criteria: (i) address the homeless population as defined in Table 1; (ii) identify barriers, enablers and interventions encountered when accessing medication and health services; (iii) focus on the experiences of individuals experiencing homelessness in healthcare settings and (iv) include qualitative accounts from members of this community and/or healthcare professionals involved in their care. There were no restrictions placed on the gender, sex, ethnicity or immigration status of participants in the studies.

**Table 1:** The Population, Interest and Context (PICo) framework used for this study [21].

Population	The target population for this study was people experiencing homelessness. Although there are many definitions of homelessness, the focus population for this review was those who fit under the definition of ‘rooflessness’ or ‘houselessness’ as given by The European Federation of National Organisations Working with the Homelessness (FEANTSA) [11]. This population either had no shelter or was residing in a temporary or institutional setting [11].
Interest	The interest of this study focused on identifying barriers, enablers and interventions addressing inequalities among the homeless population when accessing medication and health services.
Context	Although there was no definitive setting, an outreach approach was frequently used in a healthcare context. This was regularly led by a variety of healthcare professionals aiming to provide tailored services to the homeless population.

Exclusion criteria included the following: (i) studies regarding non‐homeless populations; (ii) not addressing the barriers, enablers and interventions faced by people experiencing homelessness when accessing medication and health services; (iii) non‐qualitative original studies (e.g., quantitative studies, systematic reviews or protocols); (iv) participants under the age of 18 and (v) studies in languages other than English.

### 2.3 Search Strategy, Information Sources and Study Selection

A systematic literature search was performed in October and re‐ran in December 2023 across four databases: Medline (OVID), Embase (OVID), CINAHL and Scopus. Medical subject headings (MeSH terms) were used to create the search, with Boolean operators (AND/OR) and truncation applied to refine search hits; the search strategy was developed by two members of the research team (T.B. and A.R.‐B.), alongside a medical librarian. The search strategy can be found in Supporting Information S1: Appendix B. A search of the grey literature was also performed, using Google Scholar. There were no restrictions or limits applied.

All search records were converted into the database EndNote (version X9) for ease of management; this included handling search results, removing duplicates and performing screening. The titles and abstracts of all papers obtained were reviewed independently by two authors (T.B. and A.R.‐B.). Full texts were retrieved for articles that met the inclusion criteria for further evaluation, and for those that could not be rejected without certainty. Full‐text articles were screened independently by two authors (T.B. and A.R.‐B.), whereas a third author (K.M.) was available to resolve disagreements by discussion if they arose, although they did not. The PRISMA flowchart was used to organise the studies, including the exclusion of papers that did not meet the inclusion criteria.

### 2.4 Reading, Data Extraction and Quality Appraisal

Two authors (T.B. and A.R.‐B.) closely read and re‐read the included studies to ensure close familiarity with the work. Data extraction was performed independently by T.B. and reviewed by A.R.‐B. using a customised data extraction tool informed by previous research. Extracted data included the following: context of the study; research methodology; participant characteristics; healthcare barriers, enablers and interventions; interpretation of data and study limitations. Quality appraisal was conducted by researcher T.B. and reviewed by A.R.‐B. using the Joanna Briggs Institute (JBI) critical appraisal checklist for qualitative research [22]; each paper was interrogated using the checklist, where 10 questions were ‘asked’ of the original research to (i) assess the methodological quality and (ii) determine the extent to which a study addressed the possibility of bias in its design, conduct or analysis. No studies were excluded on the grounds of quality.

### 2.5 Analysis and Interpretive Synthesis

**Figure 1:**
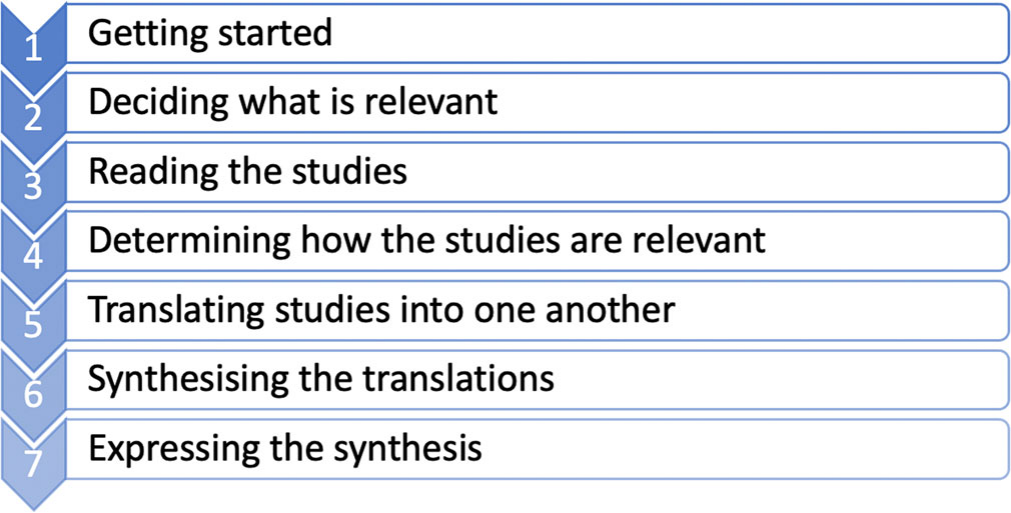
Noblit and Hare's seven‐step process for meta‐ethnography [23].

A meta‐ethnographic approach was used for this review, as determined by the seven phases of meta‐ethnography by Noblit and Hare (Figure 1) [23]. This approach of qualitative evidence synthesis is now widely used in health research [24] and uses processes of reciprocal translation to move beyond the initial reported outcomes from the primary studies and provide a more comprehensive understanding of a subject [24].

Quotations (termed ‘first‐order constructs’ taken from the primary studies) and the original author's interpretations (‘second‐order constructs’) were extracted from the studies [23]. They were compared to determine how they were related and translated into one another (reciprocal translation); this involved chronological comparison of first‐ and second‐order constructs one paper at a time to develop new interpretations [24]. This enabled the development of themes (‘third‐order constructs’) and sub‐themes as part of this interpretive synthesis.

The third‐order constructs seek to ‘go beyond’ the original interpretations of the primary studies to derive a deeper analytical understanding of the topic of medicines and healthcare inequalities for people experiencing homelessness. For the purpose of this work, the third‐order constructs have been termed ‘overarching themes’ and ‘sub‐themes’.

## 3 Results

### 3.1 Search Results

**Figure 2:**
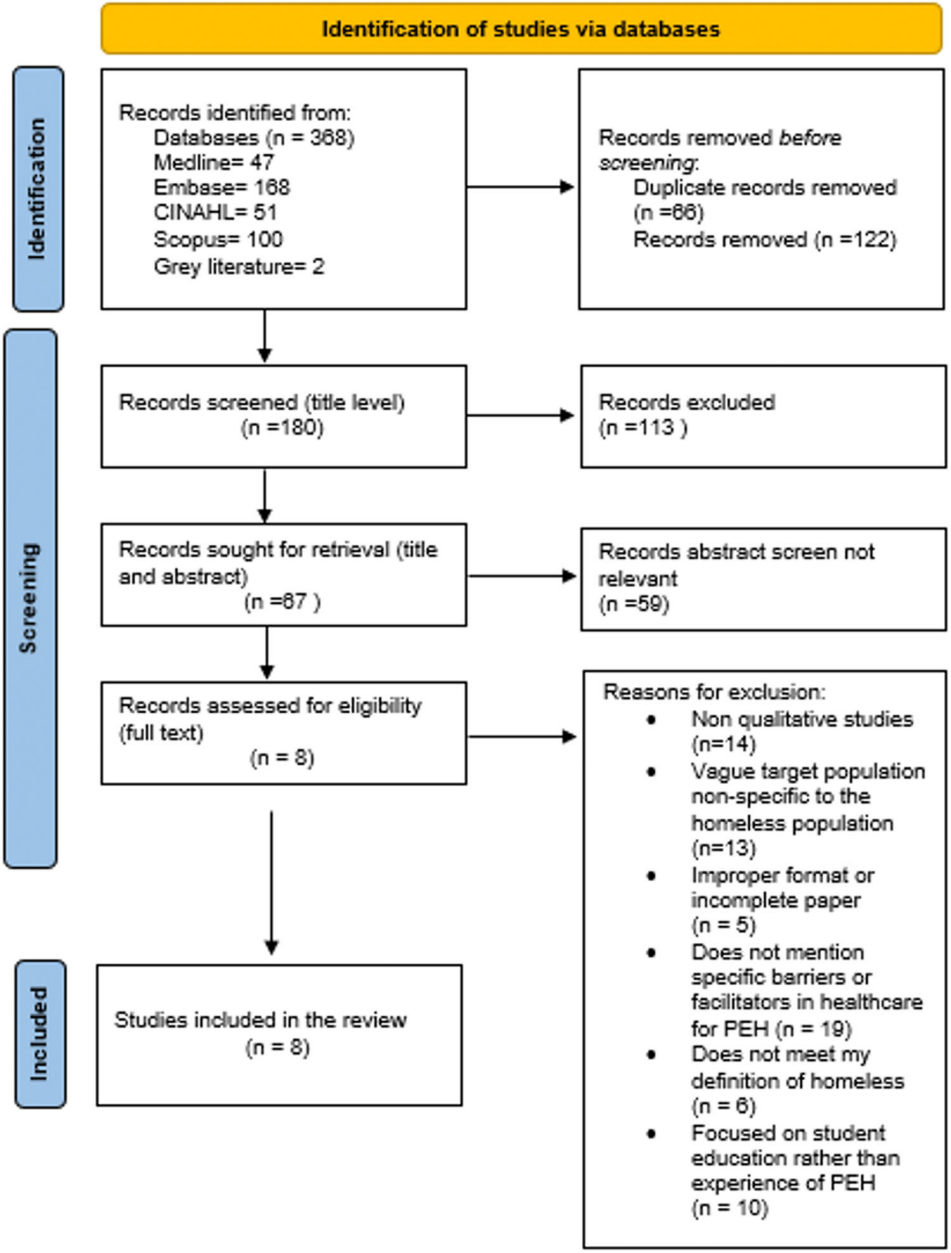
PRISMA flowchart for study selection.

A combined result of 368 papers were screened from databases (Medline [OVID], Embase [OVID], Scopus and CINAHL) and grey literature searches. Through comprehensive screening, eight studies remained. Figure 2 demonstrates the PRISMA flowchart for the study selection process, including reasons for study exclusion.

### 3.2 Study Characteristics

The eight studies included in this meta‐ethnography had publication dates that ranged from 2018 to 2023. The settings of the included studies varied in terms of geographical location and income of each country; participants were diversely represented, from low‐income cities such as Dhaka, Bangladesh [25], and Johannesburg, South Africa [26], as well as higher income nations such as the United States and the United Kingdom [27, 28, 29, 30]. Although the majority of the papers included a combination of quotes from healthcare professionals and people experiencing homelessness, two papers exclusively addressed the experiences of community pharmacists [27] and the homeless population [29]. Concerning the methodological approaches employed, semi‐structured interviews were the most popular; however, two studies incorporated surveys [27, 31]. Few studies solely examined the barriers and enablers that people experiencing homelessness encountered when accessing medication and healthcare services in isolation. Instead, it was more common for the studies to focus on further developing specific healthcare services that were offered to people experiencing homelessness; for instance, Bazzi et al. investigated patient–provider perspectives on low‐antiretroviral pre‐exposure prophylaxis (PrEP) for HIV prevention among homeless persons who inject drugs [31]. Table 2 provides a more detailed description of the study objectives, target populations and key points.

**Table 2:** Study characteristics.

References	Study design	Location and setting	Aim	Study target population	Staff involved	Study summary
1. Carmichael et al. [32]	Interviews were conducted with 69 participants. The interviews were audio recorded, transcribed and translated by researchers in both the local language and English.	Four European countries: Spain (Madrid), the United Kingdom (Norfolk), Greece (Athens and Piraeus) and Austria (Vienna). In private/semi‐private settings such as communal spaces or offices.	The aim of the study was to better understand the specific healthcare needs of the homeless population and the barriers and facilitators associated with accessing health services.	Study population; people experiencing homelessness including individuals experiencing literal rooflessness, residing in facilities or services and living in insecure or inadequate housing. This group comprised 35 participants: 13 women and 22 men. Ages ranged from 25 to 71 with a mean age of 50.	The research involved staff working in homelessness‐related support services such as social workers, as well as frontline health and social care professionals. There were a total of 15 professionals working in social care and homelessness support services and 19 healthcare professionals.	The study addressed significant health problems and barriers to healthcare PEH encountered. Healthcare barriers included lack of formal identification, absence of health insurance and language barriers; this was predominant in Greece and Austria where large migrant and refugee populations resided. The study also highlighted the lack of tolerance towards missed appointments and ‘non‐standard’ behaviours within mainstream health services, leading to exclusion or prolonged wait times for care. Facilitators to healthcare access included tailored services, outreach interventions and the involvement of trusted professionals.
2. Jagpal et al. [29]	Comprised focus groups with homeless persons utilising a topic guide developed by the research team.	England, UK Communal areas within the temporary shelter accommodation.	The study aimed to conduct public involvement sessions to inform a proposal to develop a novel, patient‐centred clinical pharmacy service for homeless persons.	Persons currently experiencing homelessness were the study's focus population. Nine participants were a part of the study: two females and seven males.	The staff involved researchers and two of the study authors directing focus group conversations.	The study explored the potential benefits of clinical pharmacy services for homeless individuals in the United Kingdom. People experiencing homelessness reported poor medical education and understanding of prescribed treatments. HCPs highlighted the need for tailored services reaching out to homeless populations and the importance of building patient–provider relationships through trust. The authors also suggested pharmacists have larger roles in providing healthcare services to homeless populations.
3. Bazzi et al. [31]	Surveys were used to assess the socio‐demographics of PEH. Qualitative interviews were then conducted to explore key domains around drug use, health concerns, HIV perceptions and experiences using the pre‐exposure prophylaxis programme (PrEP).	Boston, USA. The Boston Health Care for the Homeless Program (BHCHP)	The study aimed to determine the essential elements of the programme that both participants and providers thought were most beneficial in encouraging the use of PrEP. An additional objective was to provide guidance for future studies and programmatic initiatives involving PrEP delivery for people who inject drugs experiencing homelessness.	The target population had to be current or former homeless patients of BHCHP's PrEP programme as well as having injected drugs in the past month. Among 21 participants, 15 were male; the median age was 36 and 76% were currently taking PrEP. All participants had used heroin and/or fentanyl in the past month, whereas 95% had used methamphetamine and 90% used cocaine among other drugs.	HCPs included prescribers, patient navigators and outreach workers as well as research staff.	The PrEP programme was perceived as supportive by both participants and providers. In particular, on the same day, PrEP protocol was valued for limiting the need for patients to schedule and show up for several appointments before starting PrEP. The low‐threshold PrEP programming implemented by BHCHP integrated laboratory testing and other PrEP services into the community as opposed to requiring patients to enter clinical settings. This had a significant impact on patients who felt stigmatised by mainstream practices. Additionally, the PrEP service acknowledged the benefits of peer experiences and word of mouth to educate and recruit potential patients.
4. Johnston, McInerney, and Thurling [26]	Involved three focus groups, each with six homeless persons who access services provided by the church.	Braamfontein Johannesburg, South Africa Trinity Health Services (THS) based in an inner‐city church	The purpose of the study was to explore the reasons why PEH came to THS and what their experiences were when they sought healthcare services. To ascertain the worth of a student‐run clinic (SRC) to the community, it was necessary to gather patient feedback.	The focus of the study was on people experiencing homelessness. There were a total of 18 participants: 15 men and three women.	Pharmacy students and medical students ran the clinics supervised by doctors and pharmacists.	The paper underlined three emerging themes: homelessness affecting health, requirements for health and how the SRC helps to meet these needs. Homelessness was found to negatively impact health due to limited access to food, ablution facilities and shelter. Sexually transmitted infections, influenza, tuberculosis and dental infections were among the common illnesses encountered by PEH. Despite the need for healthcare services, participants expressed fear of stigma and discrimination leading to avoidance of medical attention. The THS was valued for treating patients with compassion and empathy, but its services were limited and restricted being student run.
5. Smith et al. [28]	Both face‐to‐face and telephone interviews were utilised.	Northeast Scotland Specialist Homeless Healthcare Centre (SHHC)	The study attempted to investigate barriers and facilitators when relocating patients from an SHHC to conventional general practices. The Theoretical Domains Framework (TDF) was employed as a framework.	17 participants in total took part; the majority of them were male and between the ages of 30 and 48 years. Many had been homeless for more than a year and characterised their general health as ‘fair’.	19 staff participants were mostly female in various occupations; general practitioners (GP), pharmacists, nurses, substance use workers and administrative staff.	The study identified several barriers to relocation, including concerns about continuity of healthcare, apprehension about meeting new staff and potential stigma. It was recommended that staff working in general and speciality practices provide proactive referrals to additional services and exchange specialised knowledge with one another, to support individuals who had recently relocated. The paper recommended establishing peer support networks and individualised plans to aid relocation processes.
6. Johnsen, Cuthill, and Blenkinsopp [30]	Face‐to‐face interviews were conducted.	Glasgow, UK Outreach services provided on the streets and through local homelessness facilities.	The study aimed to report the experiences of patients and stakeholders regarding an outreach service, which is known as the Pharmacy Homeless Outreach Engagement Non‐Medical Independent Rx (PHOENIx), delivered by pharmacists. The programme had been implemented for several years, but it recently shifted from being primarily health clinic‐based to including outreach services.	Of the interviewees with experience of homelessness, 33 were men and seven were women. Although ages ranged from 19 to early 80s, the majority of participants were aged between 30 and 40 years.	A local charity: Simon Community Scotland collaborated with a team of three pharmacists with independent prescribing qualifications.	The paper focused on the effectiveness of an outreach service delivered by pharmacists to homeless individuals. The results indicated that the programme was effective in locating and interacting with patients who were unwilling or unable to use the current healthcare services. The outreach assisted in removing obstacles to accessing healthcare, enabled prompt diagnosis and medication prescriptions and capitalised on patients determined to take care of their own needs. The informal, flexible and patient‐centred approach gave rise to optimism regarding better health outcomes for this extremely vulnerable population.
7. Tune et al. [25]	In‐depth interviews and group discussions were organised with street dwellers.	Three city streets in Dhaka, Bangladesh	The purpose of the study was to investigate the health‐related behaviours of Dhaka City street dwellers as well as their experiences with illness and their interactions with the official health systems.	The target population comprised 40 street dwellers (defined as those who sleep on the streets and open places), including 22 men and 18 women. The age of the street dwellers varied from 18 to 75 years, with the majority being male (55%), landless (95%) and uneducated (65%).	Key information interviews were conducted with six service providers and three policymakers.	The findings highlight gaps in practice and policy despite government efforts to create an inclusive and universal healthcare system. The study reported that street dwellers frequently visit retail pharmacies for advice and treatment when they are sick, that they are reluctant to go to formal facilities until they are forced to by a serious illness or injury, and that there is no efficient way for the formal health systems to get in touch with them. One significant barrier identified was the lack of an identity card that was often required by healthcare providers to provide free treatment. The authors suggested targeting this vulnerable population with tailored services to provide convenient, low‐cost healthcare.
8. Paudyal et al. [27]	Authors mailed questionnaires and a multiple‐choice quiz to community pharmacists.	England and Scotland community pharmacies	To investigate the behavioural determinants, extent of training and experiences of community pharmacists in managing and counselling the homeless population.	PEH accessing healthcare at community pharmacies were partial targets for the study adjacent to community pharmacists.	Community pharmacists were primarily the target population; however, PEH remained the focus of the study. There were 322/1951 (16.1%) responses in all, with 157 (49.5%) being from female colleagues.	According to the study, pharmacists felt unprepared to identify homeless patients and offer individualised guidance and support. However, most participants were aware of how homelessness affects people's health, happiness and ability to take their medications. The findings of the study implied that community pharmacists could benefit from appropriate training and guidelines to better their confidence and abilities in offering proactive support and guidance to homeless patients. Paudyal et al. proposed incorporating homelessness into undergraduate education for healthcare students.

Abbreviations: BHCHP = Boston Health Care for the Homeless Program, HCP = healthcare professional, PEH = people experiencing homelessness, PHOENIx = Pharmacy Homeless Outreach Engagement Non‐Medical Independent Rx (prescribing), PrEP = pre‐exposure prophylaxis, SHHC = specialist homeless healthcare centre, SRC = student‐run clinic, TDF = theoretical domains framework, THS = Trinity Health Service.

### 3.3 Quality Appraisal

Quality appraisal was carried out on all eight studies included in this review (Table 3, Supporting Information S1: Appendix C). In accordance with the JBI criteria, the studies were assigned a quality bias percentage score. The quality appraisal scores ranged from 60% to 80%, with a mean quality score of 68.75% demonstrating that the included studies' methodological quality was generally good. From the eight studies, Johnsen, Cuthill, and Blenkinsopp [30] had the highest quality score (70%), whereas Jagpal et al. [29] and Bazzi et al. [31] had the lowest quality scores (60%).

**Table 3:** Critical appraisal of the methodological quality of the included studies.

References	Quality bias score										Total score	
	1	2	3	4	5	6	7	8	9	10	(*n*)	(%)
Carmichael et al. [32]	Y	Y	Y	Y	Y	N	U	U	Y	Y	7/10	70
Jagpal et al. [29]	Y	Y	Y	Y	Y	N	N	N	U	Y	6/10	60
Bazzi et al. [31]	Y	Y	Y	Y	Y	N	N	U	N	Y	6/10	60
Johnston, McInerney, and Thurling [26]	Y	Y	Y	Y	Y	N	N	N	Y	Y	7/10	70
Smith et al. [28]	Y	Y	Y	Y	Y	N	N	N	Y	Y	7/10	70
Johnsen, Cuthill, and Blenkinsopp [30]	Y	Y	Y	Y	Y	N	Y	U	Y	Y	8/10	80
Tune et al. [25]	Y	Y	Y	Y	Y	N	N	U	Y	Y	7/10	70
Paudyal et al. [27]	Y	Y	Y	Y	Y	N	N	U	Y	Y	7/10	70
											Average = 68.75	

Abbreviations: N = no, U = unclear, Y = yes.

### 3.4 Findings: Reporting Outcomes, Synthesising Translations and Developing Themes and Sub‐Themes

**Figure 3:**
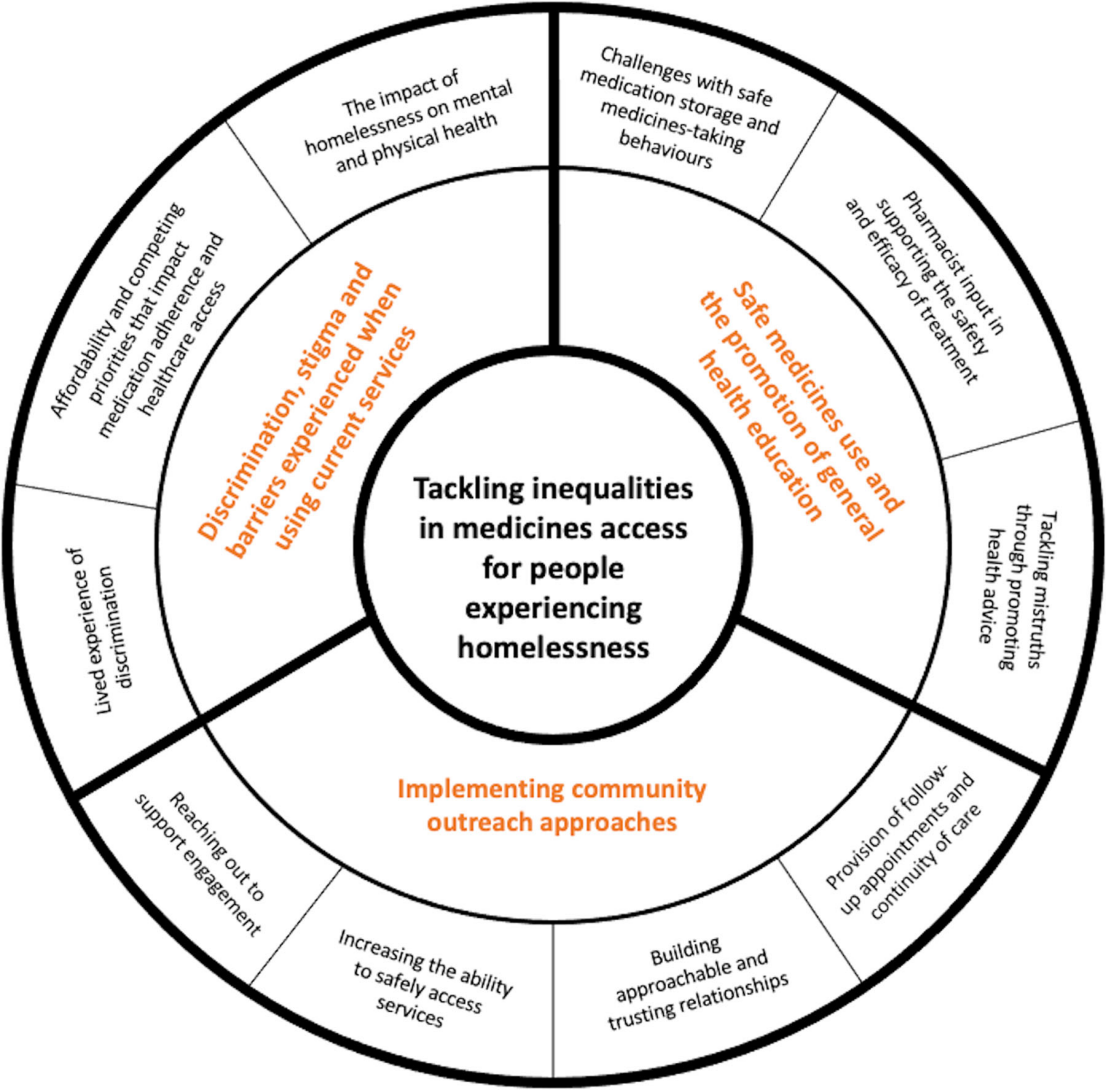
Developed themes and sub‐themes for tackling inequalities in access to medicines for people experiencing homelessness. The inner band on the diagram (orange text) represents the three overarching third‐order constructs (themes) developed by this review, whereas the outer band (black text) details the subsequent sub‐themes.

**Figure 4:**
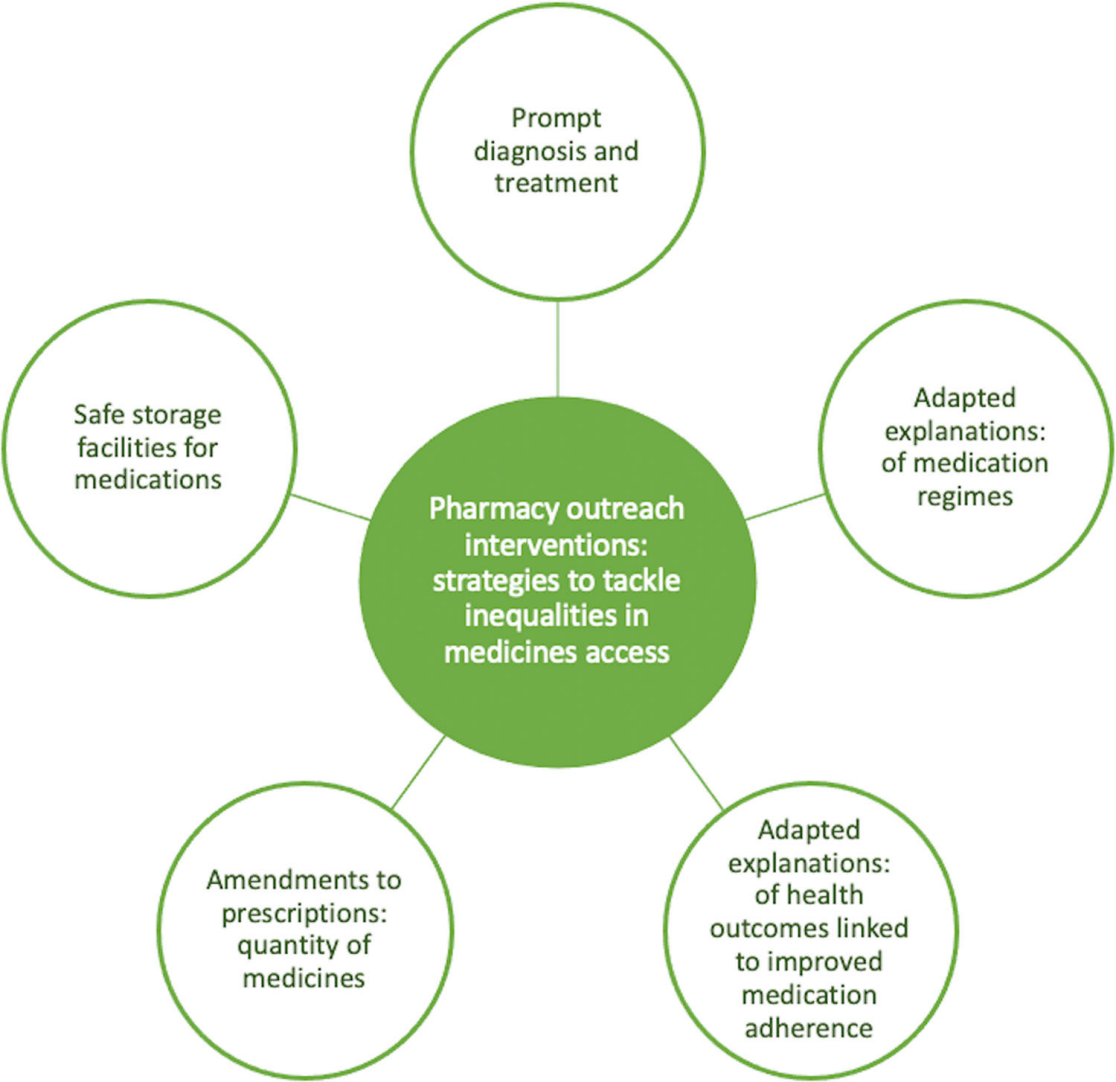
Pharmacy outreach interventions: strategies aiming to tackle inequalities in access to medicines for people experiencing homelessness.

Across the papers included in this meta‐ethnographic systematic review, there were three third‐order constructs developed (termed ‘themes’). All three themes appeared key when concerning the barriers, enablers and interventions addressing the inequalities people experiencing homelessness encounter (outlined in Figure 3). As is typical in a meta‐ethnography, each theme is presented in a table; these tables showcase examples of direct quotations (first‐order constructs) from study participants, the authors' interpretations from the original findings (second‐order constructs) and our interpretation as themes (third‐order constructs) and sub‐themes, moving beyond the findings of the original study. The three themes centred on (i) discrimination, stigma and barriers experienced when using current services (Table 4); (ii) safe medicines use and the promotion of general health education (Table 5) and (iii) implementing community outreach approaches (Table 6). Each theme, and subsequent sub‐themes, will now be discussed in turn. An overarching summary, detailing examples from pharmacy outreach interventions that were deemed to be most effective for people experiencing homelessness can be found in Figure 4.

**Table 4:** Theme 1: Discrimination, stigma and barriers experienced when using current services.

Overarching third‐order construct (termed ‘theme’) developed by the research team	Third‐order sub‐theme developed by the research team	Second‐order constructs (interpretations made by the original study authors)	First‐order construct (quotations taken directly from participants of the original studies)
Discrimination, stigma and barriers experienced when using current services	Lived experience of discrimination	Healthcare access and utilisation [30]	*was once sitting in a doctors’ surgery waiting to see a doctor with my wife, now ex, and someone else came in and the receptionist apologised to her for there being two drug users in the waiting room. In front of us!* [30] (Authors have deliberately anonymised participants; Pseudonym Ewan PEH)
		Accessing healthcare services [26]	*I'm HIV [human immunodeficiency virus] positive, they [healthcare professionals] don't trust, like the medication I'm taking is for 30 days, they don't trust me. If I get lost one of the, my ARVs [antiretrovirals] it's like I've sold it, you see*. [26] (Participant 2, Unemployed male)
			*Why are you not bathing? and "Why are you smelling?'"*[26] (Participant 1, Unemployed male)
			*I was afraid to go to the public hospital because I know how they treat people*. [26] (Participant 5, Unemployed male)
		Beliefs about consequences [28]	*A lot of them feel if they go to a mainstream surgery they're classed as a, they're treated as a second class citizen*. [28] (Staff 9, pharmacy)
		Social stigma [32]	*Some of them have not even been allowed into GP surgeries because they do not correspond to the ideal patient image of "freshly showered", "comes to the appointment well‐groomed and on time", "has everything you need", but are perhaps not freshly washed, are perhaps slightly drunk or more heavily drunk at times, are perhaps loud, are perhaps unpleasant … [they] notice that they are looked at strangely, that they are either not treated or are treated much later*. [32] (Social worker, Austria)
			*It's a real second‐class citizen system for homeless people … There's an expectation that the patient is usually more needy and drug seeking. So, every presentation usually comes back to a behaviour or addiction illness as the diagnosis, rather than pursuing it*. [32] (Nurse, UK)
			*The main barrier [in accessing health care services] is unfortunately created by us, the people who are homeless because it is very shameful to say that I have no home*. [32] (48‐year‐old female, homeless hostel, Spain)
		Accessing healthcare services [26]	*They (clinics and hospitals) are avoiding homeless people to give them such care, or to give them support in their rights (to healthcare)*. [26] (Participant 2, Unemployed male)
		Health systems experiences: long waiting time, no identity card and no services for police cases [25]	*… it is difficult to stand in a queue in the public hospital, there is too much crowd …. I faced huge trouble … once I visited a public hospital and it took around 40 minutes in the queue … so I do not want to go there anymore*. [25] (Adult woman)
			*The health policy is common for everyone. To my knowledge, there is no specific policy for the street dwellers, maybe no one even thinks of the necessity of any policy for them … nutrition and health policies are needed for them*. [25] (High government official)
			*Once the ferry ghat leader beat me and my legs and head were injured … when I visited the public hospital, they asked for my identity and address! … they did not provide services to me … provider told me to visit the police station and file a case against the leader … After that we try not to tell them that we were abused, rather we would tell that it was an accident*. [25] (A reproductive‐age woman, mother of two children)
	Affordability and competing priorities that impact medication adherence and healthcare access	Preventative healthcare as ‘non‐priority’ [32]	*The priorities of a person living in a house can be taking care of themselves, going to the doctor … but when you are living on the street, your priority is to eat something and not have your belongings stolen*. [32] (Male aged 55, residing in a homeless hostel, Spain)
			*Their eating habits on the street are basically whatever they can get their hands on. So, let's say a diabetic, a heart patient or a person with high cholesterol … they may not have access to food that is beneficial to their condition*. [32] (Psychologist, Greece)
		Practical and financial barriers to healthcare access [32]	*Most of the time, they say they will send the information to your mobile, but you do not have a mobile … or they will send the letter to your house, but you do not have a house*. [32] (Female aged 49, residing in a homeless hostel, Spain)
		Drug shops, formal facilities and structural barriers to access services free of cost [25]	*I will not go to the public hospitals. They do not provide medicines, they charge a huge amount of money for diagnostic tests … we cannot even afford our food, so why should we bother for treatments?* [25] (Elderly man)
			*I prefer to go to the pharmacy/drug shops, when I have some money … But when I have no money, I try home remedies to recover from my disease, for example, I eat ‘telakochu pata’ (kind of leaf) to recover from diabetes instead of going to the doctor …* [25] (Elderly street woman)
			*… they spend their money on buying medicines from the drug shops. But, when their illness doesn't get better, they come to visit us in the hospital … and at that time they cannot afford the cost*. [25] (Service provider, government medical college hospital)
			*For blood test, I needed BDT 500 and for ultrasonography BDT 300, which summed up to a total of BDT 800 only. I don't have any source to get this huge amount of money … eventually, I couldn't continue my treatment*. [25] (A reproductive‐age woman)
	The impact of homelessness on mental and physical health	Impact of living conditions [32]	*Absolutely, homelessness does [have an impact on health]. Not only on mental health but also physical health … Because people are constantly moving. Whether it's cold, warm, or sub‐zero, they're constantly outside and of course, that affects the body … When something hurts, many people solve it with alcohol*. [32] (Male aged 47, supported accommodation for formerly homeless people, Austria)
			*It's very hard not to have a place to live … People who live on the street can catch a lot more diseases, you know, due to not having good health, due to not having a clean home*’. [32] (*51‐year‐old male, homeless hostel, Spain)*
		Prevalence of poor health among PEH [32]	*It was really, really bad, not so much anything physical, just me in myself … I was sleeping rough, and yeah, wasn't looking after myself, didn't have any interest in anything at all*. [32] (34‐year‐old male, supported accommodation for formerly homeless persons, UK)
		Support needs of PEH [32]	*People are mentally ill, and their conditions are not treated, but it is not that they are not treated because there is no help available, but because people are simply so ill that they cannot accept any help*. [32] (Social Worker, Austria)
		Preventative healthcare as ‘non‐priority’ [32]	*[Health‐related issues] are usually left until things have got a lot worse … So when we do see chest infections, they are well‐established, and when we see wounds, they are well‐established, and when we see people with drug and alcohol issues, their addictive behaviours are well‐established*. [32] (Nurse, UK)
		Case finding and engagement [30]	*A lot of them are not registered with a GP or have other health problems … or generally they are just so chaotic that they're not going to be going to get their prescriptions regularly, or they just don't care about their health and so they're just not making that effort to be getting their prescriptions … Then if [the service] team wasn't there, then these people would be just falling through the cracks. They wouldn't be engaging with anyone at all*. [30] (Stakeholder 6)
			*I have trouble addressing folk … I think my self‐worth's been knocked that much that I don't think I'm worthy now, you know …* [30] (Authors have deliberately anonymised participants; Pseudonym Geoff PEH)
		Intentions [28]	*A number of people who I suppose I've worked with over a period of time would probably rather just stay there because they know it and it's, you know, the people and it is probably a hassle to have to go and find a GP practice and go along and fill in forms and do it all*. [28] (Staff 7, mainstream practice)
		Beliefs about capabilities [28]	*I think the self‐esteem and the confidence and, you know, kind of that element of it takes so much longer to build back up in the person*. [28] (*Staff 1, SHHC*)
		Negative experiences [27]	*Understandably many homeless patients feel threatened in their situation and often take their anger out on staff in pharmacies trying to help*. [27] (*46‐year‐old female pharmacist*)

Abbreviations: PEH = people experiencing homelessness, SHHC = specialist homeless health centre.

**Table 5:** Theme 2: Safe medicines use and the promotion of general health advice.

Overarching third‐order construct (termed ‘theme’) developed by the research team	Third‐order sub‐theme developed by the research team	Second‐order constructs (interpretations made by the original study authors)	First‐order construct (quotations taken directly from participants of the original studies)
Safe medicines use and the promotion of general health advice	Challenges with safe medication storage and medicine‐taking behaviours	Shelter and belongings [26]	*The basic thing is somewhere to sleep and where my things such as my CDs [controlled drugs] can be kept safe … You can't keep a prescription of medication for more than 3 days without losing it*. [26] (Participant 1, Unemployed male)
		Health‐related outcomes [30]	*They gave me a bit of stern advice. I didn't actually realise that the likes of paracetamol can kill you. I didn't actually realise that after ‐ I think they said ‐ 10 or 11 you can actually overdose from it.* [30] (Authors have deliberately anonymised participants; Pseudonym Donald PEH)
		Additional PrEP prescribing supports [31]	*I basically live out of a backpack [without] safe places to keep a 30‐day script … If my backpack comes up missing, they have another 21 days for me. It prevents that lapse, promotes my continually taking it, and gives me a little breathing room*. [31] (Michael, a 44‐year‐old man)
			*If it wasn't for the [PrEP storage facility], I wouldn't be taking it daily*. [31] (Participant not specified)
			*We always ask [patients] if they're concerned about losing their meds. That answer's always yes … For starting PrEP, that's one of our first questions. Then, “Where do you want us to hold onto it? Where are you most likely going to see us every day?” As long as we have that conversation, generally, people leave PrEP with us*. [31] (Clinician, not specified)
			*When we can leave our meds here [in PrEP storage facility], there's really no excuse to not take it daily*. [31] (Participant not specified)
		Positive experiences [27]	*Diabetic patient of NFA [no fixed abode] prescribed insulin ‐ nowhere safe to store pens, constantly mislaid/stolen. Got Rx changed to weekly dispense and supply kept in pharmacy fridge*. [27] (46‐year‐old female pharmacist)
	Pharmacist input in supporting safety and efficacy of treatment	Health‐related outcomes [30]	*My Prozac, they're [GP surgery is] not really helping me … What [the pharmacist] suggested was … there's a medicine out, it's two‐in‐one. It can help my blood pressure, and it can also help relax my muscles, or whatever …* [30] (Authors deliberately anonymised participants; Pseudonym Sean)
			*They're so nice and non‐judgemental, and give off the impression that they genuinely want to help, which, in the medical profession is quite rare, because they're so busy*. [30] (Authors deliberately anonymised participants; pseudonym Geoff)
			*They told me a wee bit, in layman's terms, which the doctor's never, ever sat down and told me*. [30] (Authors deliberately anonymised participants; Pseudonym Malcom)
			*… Aye, because the doctors … they'll go like that, "Well, you've got cirrhosis of the liver", and then they leave it like that … They just leave you there with that thought stuck in your head, and you're like that, "Oh, shit, cirrhosis. Wait a minute, that's dodgy. Am I going to die?" So, they [pharmacists] can answer those questions …* [30] (Authors deliberately anonymised participants; Pseudonym Malcom)
			*They talked me into going on Methadone … [Otherwise] I would have continued losing weight and buying street junk, any, whatever poison it is these swine are putting in it, and, with the hectic lifestyle, you don't eat enough as it is. I would have continued to deteriorate health‐wise … My health has took a few steps better*. [30] (Authors deliberately anonymised participants; pseudonym Geoff)
			x*So, if it wasn't for the team issuing those prescriptions and seeing these patients regularly and doing the blood pressure checks and referring these people on, I don't think anyone else would be able or willing to do it. So, the most kind of noticeable and tangible difference is … patients that weren't taking their medicines before, or weren't engaging with services are doing so because of them*. [30] (Stakeholder 6)
		Positive experiences [27]	*Discussing coming off both opioids and “legal highs” with a patient who had a lot of success in doing so alongside my support and is now in work*. [27] (28‐year‐old male pharmacist)
		Understanding prescribed treatments [29]	*… when I was in prison I started a new medication but, I've not had a chance to speak to anybody about the medication or potential side effects, whatever, whereas if there was a pharmacist there at the time, that would've helped a lot*. [29] (Male aged 50)
			*… if you've got a pharmacist to talk to, they can either back up what the doctor says or back up what you're saying and then maybe they can go to the doctor. That would be good*. [29] (Male aged 50)
			*When they put me on the medication in jail that was an anti‐psychotic as well. So, I mean, and if you're not the right person for that medication it can have really adverse effects. Luckily, I was the right person for it but* … [29] (Male aged 50)
	Tackling mistruths by promoting health advice	Health‐related outcomes [30]	*They've encouraged me to give a toss, basically, about my wellbeing, you know, because I wasn't really … I would have continued on my merry way, and there is only two ways out when you are a drug user. Death is one*. [30] (Authors have deliberately anonymised participants; Pseudonym Geoff PEH)
		Prevailing ailments [26]	*I will wish that it can be also more of a counselling in terms of sexually transmitted disease … because in the street I don't think they exercise protection*. [26] (Participant 5, Unemployed male)
			*During winter it's whereby we experience a high number of death rates on the street, due to the fact that people, they don't know their health state*. [26] (Participant 4, Unemployed male)
		Community‐driven PrEP education [31]	*I thought [PrEP] was only for gay guys [and] sexual activity, and that threw me off and made me not interested in it*. [31] (Brandon, a 34‐year‐old male)
			*There's a lot of misinformation about PrEP from earlier marketing, and stigma that it's for people with HIV … To design different materials, we had an artist and little catch sayings … so we were a part of designing new stuff, and I've seen it around [the city]*. [31] (Michael, a 44‐year‐old male)
		Prevalence of poor health among PEH [32]	*Respiratory issues, vascular issues, trench foot issues … that's the majority of the people that I see … A lot of bacterial endocarditis … and a lot of bacterial neuropathy, from sleeping rough. Things like Hepatitis C, a lot of people have*. [32] (Nurse, UK)
			*People who have problems with alcohol and heart diseases usually come here to the centre. There are a lot of liver diseases, poorly controlled diabetes, and associated problems such as amputations and vision loss*. [32] (Female aged 50, residing in homeless hostel, Spain)
			*If they're injecting, ulcerated legs do come up quite a lot, and they can get infected … Yeah, addiction, alcoholism, mixing their prescribed medication with alcohol, that's a big thing*. [32] (Support worker, UK)

Abbreviations: PEH = people experiencing homelessness, PrEP = pre‐exposure prophylaxis.

**Table 6:** Theme 3: Community outreach approaches.

Overarching third‐order construct (termed ‘theme') developed by the research team	Third‐order sub‐theme developed by the research team	Second‐order constructs (interpretations made by the original study authors)	First‐order construct (quotations taken directly from participants of the original studies)
Implementing community outreach approaches	Reaching out to support engagement	Case finding and engagement [30]	*… if [the service] team wasn't there, then these people would be just falling through the cracks. They wouldn't be engaging with anyone at all*. [30] (Authors have deliberately anonymised participants; Stakeholder 6)
			*They frequently didn't turn up or didn't do what was asked of them and you still stick with them. I can see evidence of that in terms of that they [the pharmacists] persevere with that*. [30] (Authors have deliberately anonymised participants; Stakeholder 5)
		Health‐related outcomes [30]	*So if it wasn't for the team …seeing these patients regularly … I don't think anyone else would be able or willing to do it. So, the most kind of noticeable and tangible difference is … patients that weren't taking their medicines before, or weren't engaging with services are doing so because of them*. [30] (Authors have deliberately anonymised participants; Stakeholder 6)
		Screening, diagnosis of health conditions and prescribing of medicines [29]	*They seem to be the sort of people that don't go to doctor's appointments, don't go and see doctors, don't go and get it taken care of. Cause they're living their day to day routine on the streets … they're developing multiple physical or mental problems, but they (also) never search for any help so, a pharmacist going out to them, they'd be getting the care and attention that they should be getting but, they're choosing not to for whatever reason*. [29] (Male aged 50)
		Prospect of outreach visits by pharmacists [29]	*They (persons experiencing homelessness) don't go to a doctor … so, if you went out to them … they would probably open up to you*. [29] (Female aged 55)
		Intensive outreach and navigation [31]	*[The PrEP navigator] knows where to find me and reminds me of appointments and taking [PrEP] … I would forget if I didn't have someone behind me, and I wouldn't care; I wouldn't do any of it*. [31] (Crystal, a 46‐year‐old woman)
			*I got offered [PrEP] before, took it the first day, but then never went back. But this time I've been following through and they've been making it really easy for me because [the navigator] is always around, doing everything he can to try to help us*. [31] (Matthew, a 34‐year‐old man)
	Increasing the ability to safely access services	Involvement of non‐clinical professionals [32]	*… This means that the services have to be implemented in such a way that people can make use of them even if they have no one to take them by the hand*. [32] (Primary Care Physician, Austria)
		Practical and financial barriers to healthcare access [32]	*Most of the time, they say they will send the information to your mobile, but you do not have a mobile … or they will send the letter to your house, but you do not have a house*. [32] (Female aged 49, residing in homeless hostel, Spain)
		Healthcare access and utilisation [30]	*In [the Homeless Health Service], I've got too many pals, or enemies … If it's pals, they take me away for drink or drugs, and I end up steaming, or else I bump into somebody that I've hurt, or who's hurt me*. [30] (Authors have deliberately anonymised participants; Pseudonym Gary, PEH)
			*I feel comfortable in this building. There's not a lot of [city centre name] where I feel comfortable in, including the walk up to [the HHS], because you meet every waif, stray, and bad‐un on the way up there, and … you will bump into the wrong ones*. [30] (Authors have deliberately anonymised participants; Pseudonym Graeme, PEH)
			*It's a hell of a walk. And sometimes I bump into people on the way and don't end up making it anyway [laughs]. There's little risk of that happening when they [the pharmacists] come here [hostel]*. [30] (Authors have deliberately anonymised participants; Pseudonym Peter, PEH)
			*It's too far for them to go to [the HHS] in a wheelchair and stuff like that or with a Zimmer or any sort of walking aid. These guys can actually just come along here [in hostel] to see [name of pharmacist] …* [30] (Authors have deliberately anonymised participants; Stakeholder 3)
		Facilitating access to medicines [29]	*I've got to walk on my heel sometimes, very painful, and if there was a pharmacist down there that'd save me a trip*. [29] (Male aged 50)
			*Yeah, I've missed medication cause I, I couldn't get, cause I suffer with arthritis so, certain days I, it's a no go even walking, I can't walk*. [29] (Female aged 55)
		Low‐threshold, accessible PrEP programming [31]	*More providers need to understand how beneficial it is coming to where people are, and recognize the barriers [for] people coming into the clinic. We have to be more understanding that that is really very difficult*. [31] (Clinical provider)
		Case finding and engagement [30]	*Signposting works for some people but not all, and people who it works least for are the people who are in the most chaos and for whom priority isn't always health. … people don't always make it to the place that's been signposted*. [30] (Authors have deliberately anonymised participants; Stakeholder 8)
		Tailored approaches to service delivery [32]	*The service [mobile health care unit] was there, and they [health care professionals] offer it to you … you don't have to go looking*. [32] (Female aged 42, temporarily residing with family/friends, UK)
	Building approachable and trusting relationships	Social and professional role and identity [28]	*… we've got good relationships with them so we could use those relationships to be able to support them and find out more information about their movement from one practice to another*. [28] (Staff 1, specialist homeless healthcare centre)
		Trusting patient–provider relationships [31]	*Going out to them to engage them in conversation, respecting who they are, and saying, ‘You deserve better than this,’ which I don't think is a common phrase in these populations*. [31] (Nonclinical provider)
			*It goes way beyond the medication. It's the people that are involved …They're personable, really comfortable to be around, and [they] have never changed who they were, not once. And that's a big part of why I'm still so involved with [PrEP]*. [31] (Jennifer, a 42‐year‐old female)
			*We see [our patients] every day. They have significant distrust in the medical establishment, but they know us and trust us … So eventually, if we've established enough trust, it's easier to talk about uncomfortable [or] very private things, like [sex work] … So it's just constant engagement and creating a safe space*. [31] (Clinical provider)
			*They [patient] know everything's confidential; we're not sitting there typing everything into their [medical] record. It's just a safe space for everyone*. [31] (Clinical provider)
			*In this lifestyle, being personable goes a long way. When you're homeless and getting high, you don't trust anybody; you don't have anybody. So when somebody's taking time out of their day to try to help you, you take it seriously*. [31] (Matthew, a 34‐year‐old male)
			*[We] like learning from one addict to another, not from staff, so we can relate better. So hearing from people who are on [PrEP] is really important*. [31] (David, a 39‐year‐old male)
		Case finding and engagement [30]	*I have trouble addressing folk … I think my self worth's been knocked that much that I don't think I'm worthy now, you know … [Name of pharmacist] is just approachable … and you get the impression when you're speaking to them, they're interested in you … They're so nice and non‐judgemental, and give off the impression that they genuinely want to help, which, in the medical profession is quite rare, because they're so busy*. [30] (Authors have deliberately anonymised participants; Pseudonym Geoff PEH)
			*… you're more at ease with them [the pharmacist and street team worker] … My doctor's a great guy in many ways, and the practice nurses and pharmacists, but it's a different way of approach in here [day centre]. They make you more at ease … They spend time listening to you … I feel really good, relaxed, the way they come across*. [30] (Authors have deliberately anonymised participants; Pseudonym John PEH)
		Positive experiences [27]	‘*A homeless patient came in the pharmacy for his weekly medication pick up. He was pale and tired looking. He felt much better after we've given some snacks and water and he was grateful!*’ [27] (*27‐year‐old male pharmacist)*
		Positive patient–professional relationships [32]	‘*There was this social worker … I kept her, only her, I sent away all the others who came … instead of canned food or bread or food, she left a thermometer, painkillers, and Niflamol for my teeth. She listened to me. It was not about what she gave me, but that she listened to me*’. [32] (*Male aged 56, residing in homeless hostel, Spain)*
			‘*When I talk to drug and alcohol users, I always use the street terminology … and I dress like this for a reason, I think uniforms are a barrier … if I'm wearing a uniform, people won't engage*’. [32] (*Nurse, UK)*
		Involvement of non‐clinical professionals [32]	*We've got one young lady, she's terrified of the doctor, and she just wants you there, she'll say, ‘please hold my hand,’ and then she'll talk to the doctor because you're with her … I know how to talk to them to encourage them to tell the doctor the symptoms*. [32] (Support Worker, UK)
			*The respect that they are giving us as homeless people is one of the most important things in terms of it's one of the things that encourages us*. [32] (Participant 5, Unemployed male)
			*Our best practice was exactly this … having a person who belongs to or used to belong to this group. It is fundamental for them to feel that a person is like them, understands them, or “speaks their language”, their slang. This makes communicating with them much easier*. [32] (Nurse, Greece)
			*They [health care professionals] were very clear with me from the beginning, they care about telling me about everything that is happening to me*. [32] (Male aged 51, residing in homeless hostel, Spain)
		Peer Support and Social Influences in Engaging with Services [29]	*And when one person hears from another that they, they sent the wellbeing bus or whatever and it helps them, and they move on or they got better. Word of mouth alone is going to build trust amongst these people*. [29] (Male aged 50)
			*Suggesting recruiting people like us, who are already on the street, some of us, going to know certain people … "here mate, I'll signpost you to them."* [29] (Male aged 50)
	Provision of follow‐up appointments and continuity of care	Additional PrEP prescribing supports [31]	*As a primary care doctor, a concern starting someone on PrEP is like, who is going to [get] their labs and track them? It's a little nerve‐racking, wondering who's followed up, how many are still taking PrEP, who's due for labs? So it's nice to know that someone is keeping track and calling people if they're overdue … It's a nice backup system*. [31] (Clinical provider)
			*For the folks who just absolutely can't take [PrEP] daily, they know they can't, and they're like, “… you make sure I take them”. Those are the people we're searching out on the street …The nurses will literally deliver one pill for today, and then again tomorrow …* [31] (Clinical provider)
			*I've been taking [PrEP] without fail because every time they see me, they give it to me, and I take it*. [31] (Diego, a 38‐year‐old man)
		Community‐driven PrEP education [31]	*the best way to disseminate [information] in this community … it's something that we [providers] don't have a say [in] because we're not in that community*. [31] (A non‐clinical provider)
		Inflexible and fragmented systems [32]	*At the time of follow‐up, a homeless person is seen as “uncomfortable” for us … I say this because we will not be able to have a follow‐up, and there may be difficulties in coordinating with social workers, firstly because there is not always one in a health care centre, or because you have to refer them [the homeless patient] to another place and you don't know if that person is going to attend for any reason*. [32] (Primary Care Physician, Spain)
		Practical and financial barriers to healthcare access [32]	*Most of the time, they say they will send the information to your mobile, but you do not have a mobile … or they will send the letter to your house, but you do not have a house*. [32] (Female aged 49, residing in homeless hostel, Spain)
		Case finding and engagement [30]	*See when you get somebody who's sat there with COPD, can't walk, and has to go to [the primary care provider], or somebody who's got leg wounds which need to be treated there and then, but isn't going to [the hospital], then [name of pharmacist providing outreach service] can do that. That's such a difference …* [30] (Authors have deliberately anonymised participants; Stakeholder 1)

Abbreviations: COPD = chronic obstructive pulmonary disease, HHS = homeless health service, PEH = people experiencing homelessness, PrEP = pre‐exposure prophylaxis.

### 3.5 Theme 1: Discrimination, Stigma and Barriers Experienced When Using Current Services

This theme highlights reasons for disengagement with current healthcare services underpinned by experiences that members of the homeless community have faced, particularly around (i) lived experience of discrimination; (ii) challenges with affordability and competing priorities that impact medication adherence or healthcare access and (iii) the impact of homelessness on mental and physical health.

#### 3.5.1 Lived Experience of Discrimination

Participants often reported feeling discriminated against, sharing first‐hand accounts of condescending language and blame targeted towards them. People experiencing homelessness also shared perspectives around mistrust of healthcare professionals, which healthcare professionals themselves echoed—particularly, how common it was to stigmatise stereotypical behaviours, such as addiction, instead of pursuing accurate diagnoses. This deterred patients from approaching healthcare providers and asking for assistance due to fear of accusations and embarrassment.

I was once sitting in a doctors' surgery waiting to see a doctor with my wife, now ex, and someone else came in and the receptionist apologised to her for there being two drug users in the waiting room. In front of us! [30]

(Authors have deliberately anonymised participants; Pseudonym Ewan)

#### 3.5.2 Affordability and Competing Priorities That Impact Medication Adherence or Healthcare Access

The cost of medication served as a barrier to accessing treatment and further testing. Hospital treatments and diagnosis were often a last resort for people experiencing homelessness and by then, individuals had little money to afford the services. In countries like Bangladesh, individuals reportedly turned to traditional remedies to treat their conditions when they had no money [25]. Financial difficulties prevented members of the homeless community from maintaining a healthy diet and led to the consumption of low‐nutrient foods; this made it difficult for patients to manage conditions like diabetes and improve their health outcomes.

I prefer to go to the pharmacy/drug shops, when I have some money … But when I have no money, I try home remedies to recover from my disease, for example, I eat ‘telakochu pata’ (kind of leaf) to recover from diabetes instead of going to the doctor …. [25]

(Elderly street woman)

#### 3.5.3 The Impact of Homelessness on Mental and Physical Health

Across many studies, members of the homeless community and healthcare professionals involved in providing care discussed the impact that being homeless can have on an individual. Clear similarities were reported from studies across the globe, where it was common to see people experiencing homelessness accessing health services once physical health symptoms/illnesses were advanced or well‐established stage. The same was reported for mental health illness too, with participants attributing poor mental well‐being as a result of being homeless. One person experiencing homelessness described how this then made them feel less worthy of engaging with health services.

… I think my self‐worth's been knocked that much that I don't think I'm worthy now, you know …. [30]

(Authors have deliberately anonymised participants; Pseudonym Geoff)

### 3.6 Theme 2: Safe Medicines Use and the Promotion of General Health Advice

This theme recognised the challenges that people experiencing homelessness reported when it comes to managing their health, and the following sub‐themes highlight mechanisms to support: (i) challenges with safe medication storage and medicine‐taking behaviours, (ii) pharmacist input in supporting the safety and efficacy of treatment and (iii) tackling mistruths through promoting health advice.

#### 3.6.1 Challenges With Safe Medication Storage and Medicine‐Taking Behaviours

Across a number of studies, inadequate storage of medication was reported to affect the medicine‐taking behaviours, adherence and personal safety of the individual experiencing homelessness [31]. Participants described how carrying large quantities (such as a month's worth of medicines) at one time in their backpacks, without safe storage, frequently made them easy targets for theft or resulted in lost prescriptions [27]. Another participant described how it was unattainable to storage followinstructions, especially for those who required temperature‐controlled storage. Mechanisms to tackle these challenges included offering access to storage services, and adjustments to prescription lengths/quantities to support reassurance to enable ongoing medication adherence [31]; for example, provisions of 7‐day prescriptions rather than 30‐day ones [30].

I basically live out of a backpack [without] safe places to keep a 30‐day script … If my backpack comes up missing, they have another 21 days for me. It prevents that lapse, promotes my continually taking it, and gives me a little breathing room. [31]

(Michael, a 44‐year‐old man experiencing homelessness)

#### 3.6.2 Pharmacist Input in Supporting the Safety and Efficacy of Treatment

There were instances across the included studies where people experiencing homelessness described the input from pharmacy teams as supportive [30]. Specifically, this related to pharmacists as being a source of advice to help people better understand their health conditions more broadly, such as an enhanced understanding of the impact of illicit drug use on long‐term health, as well as the potential side effects of prescribed medication. In the study by Jagpal et al., participants who were experiencing homelessness described the potential role of pharmacists in preventing and managing the side effects [29]. These participants also viewed pharmacists as a point of information to query about medications, when they did not ask the original prescriber due to time constraints [29].

When I was in prison I had an addiction diagnosis, I started a new medication but, I've not had a chance to speak to anybody about the medication or potential side effects, whatever, whereas if there was a pharmacist there at the time, that would've helped a lot. [29]

(Male, aged 50)

#### 3.6.3 Tackling Mistruths by Providing General Health Advice

A number of studies included mention of the benefits of health promotion when it came to supporting both the physical and psychological health of people who were homeless. Participants who were healthcare professionals reported higher rates of diseases like bacterial endocarditis, as well as poorer psychological health, among this vulnerable population [32]. They reinforced the message that an individualised care approach to health advice and support should be adopted when working with people experiencing homelessness; doing so appreciated the heterogeneity in each individual's experience of homelessness, interwoven with other forms of vulnerability and social exclusion (such as migrant status and substance misuse) [32]. Furthermore, particularly when it came to tackling myths and mistruths around PrEP, people experiencing homelessness reported it useful to learn about ways they can improve their own health. One participant experiencing homelessness discussed a personal experience of being involved in developing community‐driven education materials to support others in the homeless community programme [31].

There's a lot of misinformation about PrEP from earlier marketing, and stigma that it's for people with HIV … To design different materials, we had an artist and little catch sayings … so we were a part of designing new stuff, and I've seen it around [the city]. [31]

(Michael, 44‐year‐old male, experiencing homelessness)

### 3.7 Theme 3: Implementing Community Outreach Approaches

This theme highlights the scope for adopting and implementing an outreach approach to provide medicines and health services to communities facing homelessness to tackle inequity. This theme encompasses the following four sub‐themes: (i) reaching out to support engagement; (ii) increasing the ability to safely access services; (iii) building approachable and trusting relationships and (iv) provision of follow‐up appointments and continuity of care.

#### 3.7.1 Reaching out to Support Engagement

Healthcare professionals recognised the lower rates of engagement with healthcare services often associated with people experiencing homelessness. As a mechanism to address this, proactive outreach strategies specifically targeting the homeless population were emphasised as key—as opposed to expecting engagement from members of this community in existing clinics [30]. Specifically, members of pharmacy teams felt that this approach could ensure patients who would not typically be seen were able to more easily interact with medicines services.

… if [the service] team wasn't there, then these people would be just falling through the cracks. They wouldn't be engaging with anyone at all. [30]

(Authors have deliberately anonymised participants; Stakeholder 6)

Similar perspectives were echoed by healthcare professionals from other job roles (including doctors and nurses), where it was reported that members of the homeless community had lower rates of attendance at their existing clinic appointments too. Each healthcare professional group recognised the challenges around ‘erratic daily life' that may be experienced by a person who was homeless; thus, support for outreach clinics was widely discussed as a means to provide a form of health service stability [29]. Johnsen, Cuthill, and Blenkinsopp reported that this strategy was effective in encouraging patients to make lifestyle changes and, specifically, to increase compliance with prescribed medication [30].

#### 3.7.2 Increasing the Ability to Safely Access Services

People experiencing homelessness described a multitude of obstacles that may prevent them from accessing services safely. Two studies specifically referenced how current strategies of signposting to external healthcare sites do not seem to be effective in increasing accessibility for this vulnerable cohort [30]. It was expressed that adopting the approach of outreach clinics could support in overcoming such challenges, many of which were related to medical, geographic, financial and safety factors [31].

I feel comfortable in this building. There's not a lot of [name of city centre] where I feel comfortable in… because you meet every waif, stray, and bad‐un on the way up there, and … you will bump into the wrong ones. [30]

(Authors have deliberately anonymised participants; Pseudonym Graeme)

#### 3.7.3 Building Approachable and Trusting Relationships

Across the studies, there was repeated mention of the importance of establishing an inclusive and approachable patient–provider relationship. Reasons for this centred on forming trusted connections among a community that has strong connections with experiencing social isolation and discrimination, given the stigma attributed to homelessness [30]. A number of participants from the original studies remarked that the patient–provider relationships that were fostered through trust and respect were the most effective, with one example demonstrating improved medication adherence and subsequent health outcomes as a result [31]. Involving and including members of the homeless community in outreach attempts was seen as a strategy to improve communication and engagement [32].

Having a person who belongs to, or used to belong to, this group [the homeless community]. It is fundamental for them to feel that a person is like them, understands them, or “speaks their language”, their slang. This makes communicating with them much easier. [32]

(Nurse, Greece)

#### 3.7.4 Provision of Follow‐Up Appointments and Continuity of Care

The inability to effectively arrange follow‐up appointments to ensure continuity of care for members of the homeless community was a cause of concern reported by healthcare professionals [31]. This was, particularly, true for referrals made to the wider multi‐disciplinary team, such as care coordination with social workers, with authors describing systems as too inflexible and fragmented in their current format [32]. Instead, mobile outreach approaches were viewed as an opportunity to enable regular contact and follow‐up with patients being delivered directly within a community itself.

At the time of follow‐up, a homeless person is seen as “uncomfortable” for us … I say this because we will not be able to have a follow‐up …. [32]

(Primary Care Physician)

## 4 Discussion

To our knowledge, this is the first meta‐ethnographic systematic review synthesising global literature to examine the barriers, enablers and current interventions seeking to address inequality in accessing medication and healthcare services for people experiencing homelessness. Three overarching themes (third‐order constructs) were developed and centred around recognising and acknowledging the discrimination, stigma and barriers experienced when using current services; exploring safe medicines use and the promotion of general health education; and appreciating strategies to tackle such inequalities, namely through the implementation of community outreach programmes designed for people experiencing homelessness.

Wider literature has previously reported a culture of discrimination and blame in healthcare settings towards people who are homeless [26, 33]. The concepts of ‘I‐You’ and ‘I‐It’ relationships have been explored by researchers analysing interactions between medical professionals and people experiencing homelessness [34]. These findings demonstrated evidence of the power disparity and dehumanisation that patients can experience in medical environments. As a result of existing stereotypes, there was a greater likelihood that members of the homeless community felt uncomfortable and belittled during consultations, compared to their non‐homeless counterparts [6, 34, 35]. Future research should seek to examine the language and terminology used by healthcare professionals when referring to people experiencing homelessness and adopt inclusive, alternative terms approved by their members of the community. Furthermore, to challenge stigmatising attitudes towards the homeless population, researchers could seek to better include and involve the voices of the homeless in future work [36].

Establishing trusted and respectful connections between healthcare workers and the homeless population was crucial for overcoming the underlying mistrust in the current healthcare system [33, 37]. Although mistrust between the homeless community and medical professionals was identified as a barrier, it was also discovered that clinicians' experience of (or lack of) working with people experiencing homelessness was an important factor underpinning the success of these relationships [30]. Wider studies have demonstrated inadequate training opportunities provided by educational institutions for healthcare students who fail to address the needs of marginalised populations, including those who are homeless [38, 39]. Many recent studies have examined approaches to inform students about cultural competence and the inequalities that exist in healthcare; however, homeless populations were frequently absent from curricula [40, 41, 42, 43]. Future research should seek to explore and implement such learning opportunities within healthcare curricula, utilising concepts such as lived‐experience narratives [44] or co‐design [45].

A community outreach approach proved highly effective when providing healthcare services to a vulnerable population, such as people experiencing homelessness; the convenience of outreach services was attributed in these studies to reduced travel, little to no transportation costs, as well as fewer detours from the person's intended destination [29, 30, 32]. This was especially helpful for members of the homeless community with co‐morbid chronic illnesses (such as COPD or arthritis) that physically hindered their ability to travel great distances [29, 32]. However, community‐based approaches have also been attributed more broadly within wider literature to feelings of enhanced belonging within a place [46, 47, 48], connectivity with other members of their local community and fewer feelings of social isolation [49, 50, 51]; all of these broader social considerations can be viewed as complementary in a person's physical and psychological health outcomes [52, 53, 54]. Within the wider literature, increased uptake of vaccinations [55] and disease screening services [56] and reduced emergency service admissions [57] have been reported among homeless populations as a result of outreach and mobile health programmes.

Although outreach services were successful and well‐received in the included studies in this review, wider concerns have been raised regarding the responsibility of mainstream health and medicines services to enhance care quality, as well as improve accessibility, for people experiencing homelessness [4]. Echoing this, a recent scoping review suggested expanding the flexibility of healthcare systems by scheduling longer appointments for the homeless population to address complex health issues, in addition to meeting their basic needs for food and shelter [56]. Migrants and refugees have previously been reported as an over‐represented group among the homeless population, confronting additional barriers when seeking healthcare [58]. Several factors, including poverty, loss of social and cultural ties and language barriers, have impacted the health status of this minority group [58]. Although outreach programmes have been deemed an effective means to connect with members of the homeless population, future studies should seek to provide cultural services and educate staff about the additional challenges faced by migrant populations [59].

This meta‐ethnography identified barriers encountered by people experiencing homelessness when accessing medication and healthcare services and identified current interventions that attempted to lessen the disparity in accessibility. Although the approach to this research was novel, it is recognised that there are some limitations. First, as the process of conducting meta‐ethnographic reviews is primarily interpretive, the results may not fully represent all lived experiences of the homeless population when accessing healthcare or medicines services. Furthermore, the studies were restricted to adult cohorts and to those published in the English language only; these restrictions may have limited the populations studied and therefore may not have captured views of wider marginalisation, such as those of young people who experience homelessness. There was diversity included within the studies in this review; this work synthesised the current global literature base, across a range of countries. Distinctive cultural traits were not explored; however, the authors recognise that this may have the potential to influence public perception of the homeless population and consequently how people experiencing homelessness may access healthcare. Comparably, there was no emphasis on the role that immigration status, ethnicity, gender or religion played in obtaining healthcare; the research team recognised that these groups have additional challenges that may require further independent investigation [56].

## 5 Researcher Positionality and Reflexivity Statement

When conducting research on health inequity and its wider connection to experiences of homelessness, it is important to acknowledge the positionality and reflexivity of the research team. The authors recognised their privilege as not having had personal lived experience of homelessness, but act as allies in tackling inequalities within pharmacy practice and wider healthcare for marginalised communities. This systematic review and meta‐ethnography was conducted by a team based in the United Kingdom; however, it appreciated international literature on the topic. T.B. is an undergraduate pharmacy student with experience in delivering public health services; K.M. is a public health speciality registrar who has conducted a health needs assessment with the homeless community in a UK city; and A.R.‐B. is a specialist clinical pharmacist and researcher with expertise in medicines inequity for marginalised groups and has specific experience in delivering care to people experiencing homelessness.

## 6 Conclusion

Findings from this meta‐ethnographic systematic review highlighted the barriers, enablers and interventions that sought to address inequality among the homeless population when accessing medication and healthcare services across multiple countries. The implementation of community‐based outreach approaches and improved provider–patient relationships were found to be key facilitators in lowering the barriers to healthcare and medication access for people experiencing homelessness. Future research should seek to further engage with homeless populations through lived‐experience and co‐design narratives aiming to enhance care quality, as well as improve accessibility, satisfaction, equity and wider health outcomes for people experiencing homelessness.

## Author Contributions

**Tasnim Begum:** investigation; formal analysis; project administration; writing–original draft. **Kylie Murrell:** supervision; writing–review and editing; validation. **Anna Robinson‐Barella:** conceptualisation; methodology; formal analysis; supervision; writing–review and editing; visualisation.

## Ethics Statement

This work is a meta‐ethnographic systematic review synthesising data from published studies; therefore, ethical approval was not required. There was no need to undergo or seek ethical approval for this work, as it is a systematic review.

## Consent

There were no patients involved in this study. This is a meta‐ethnographic systematic review synthesising data from published studies; therefore, patient consent was not required.

## Conflicts of Interest

A.R.‐B. is affiliated with the NIHR Newcastle Patient Safety Research Collaboration (PSRC). The other authors declare no conflicts of interest.

## Supporting information

Supporting information.

## Data Availability

The data that support the findings of this study are available from the corresponding author upon reasonable request.
